# Editorial: Targeting Neuro-Immuno-Vascular Interactions in the Brain and the Periphery

**DOI:** 10.3389/fphar.2022.893384

**Published:** 2022-04-26

**Authors:** Imola Wilhelm, István A. Krizbai, Mihaela Gherghiceanu, Éva Szőke, Zsuzsanna Helyes

**Affiliations:** ^1^ Institute of Biophysics, Biological Research Centre, Szeged, Hungary; ^2^ Victor Babeș National Institute of Pathology (INCDVB), Bucharest, Romania; ^3^ Department of Pharmacology and Pharmacotherapy, Medical School and Szentágothai Research Centre, University of Pécs, Pécs, Hungary

**Keywords:** neuroinflammation, inflammasome, pain, neuropathy, ischemic stroke

Complex neuro-immune-vascular interactions play key roles in the development of both neuroinflammation (an inflammatory response within the nervous system) and neurogenic inflammation (sensory nerve-released neuropeptides inducing inflammation in different tissues). Both phenomena have a substantial importance in the pathogenesis of several diseases and might be targets for pharmacological interventions.

Therefore, the present Research Topic was designed to collect papers on the molecular mechanisms and pharmacology of pathological processes affecting neuro-immune-vascular interfaces, focusing on neurodegeneration, stroke, headache, neuropathic and other types of pain, as well as peripheral inflammatory diseases, such as arthritis or dermatitis. In addition, interactions between the peripheral and central nervous systems have also been explored.

Neuroinflammation is usually accompanied by vascular reactions, including opening of the blood-brain barrier. In the present Research Topic, Gabbert et al. (2020) describe the important role of protocadherins, especially PcdhgC3 in regulating the barrier integrity of brain microvascular endothelial cells in control and inflammatory conditions. One of the most frequent inflammatory conditions affecting brain vessels is ischemic stroke. Recanalization with tissue plasminogen activator (tPA) is the only approved agent available in this condition. The systematic review by Ye et al. (2020) identifies the possible protective effect of immunomodulators on a lethal side effect of tPA treatment, namely hemorrhagic transformation. In addition, Chen et al. (2021) found that inflammasome proteins were upregulated in neutrophil extracellular traps present in thrombi of patients with acute ischemic stroke, contributing to poor outcomes after tPA treatment.

Inflammasomes were in the focus of the work of Nógrádi et al. (2020) as well. However, they studied not vascular cells, but neurons and found that peripheral nerve injury induced inflammasome activation in motoneurons. The thiazide structure oral antidiabetic diazoxide proved to be protective in this model. Interlocking of peripheral and central processes has been investigated by Lovrenčić et al. (2020) too. They found that intranasal inflammatory reactions provoke distant intracranial changes, including headache.

Inflammatory reactions may affect the peripheral nervous system as well. Inflammatory neuropathies are characterized by leukocyte infiltration of peripheral nerves, demyelination and axonal degeneration. Therefore, inflammation and pain control in chronic neuropathies is essential to improve the outcomes of the disease. Somatostatin released from capsaicin-sensitive peptidergic sensory nerve endings leads to anti-inflammatory and anti-hyperalgesic actions at distant parts of the body, through activation of the somatostatin receptor subtype 4 (SST4). Based on this knowledge, Kántás et al. (2021) demonstrated the anti-nociceptive actions of orally active novel pyrollo-pyrimidine SST4 agonists, which are promising drug candidates in neuropathic pain.

A specific subgroup of neuropathies, immune axonal neuropathies are described in the review of Tulbă et al. (2021). These immune-mediated neuropathies occasionally accompany autoimmune rheumatic diseases, which in turn may also have cardiovascular comorbidities. These latter conditions have been explored by Mong et al. (2020), who found that arterial aging is faster in rheumatoid arthritis patients than in control subjects, and inhibiting inflammation is essential to attenuate the associated cardiovascular risk.

Finally, the paper of Szöllősi et al. (2022) reviews a symptom arising from cutaneous immune-neuronal crosstalks, i.e., pruritus or itch, which may involve the excitation of itch-sensitive neurons or damage of the itch-processing neural network.

Altogether, as a result of the common effort of the Frontiers in Pharmacology team and the five guest editors, a balanced collection of original and review papers have been included in the Research Topic. Nine articles originating from six different countries cover a wide thematic range focused around inflammatory processes affecting the central and peripheral nervous systems.
